# Testing the Implementation Framework for Behavioral and Lifestyle Interventions in Alzheimer's Disease (MOBILIZE) via the ACT Randomized Controlled Trial

**DOI:** 10.1002/alz70860_102233

**Published:** 2025-12-23

**Authors:** Dereck L. Salisbury, Feng Vankee Lin, Fang Yu

**Affiliations:** ^1^ University of Minnesota, Minneapolis, MN, USA; ^2^ Stanford University, Stanford, CA, USA; ^3^ Arizona State University, Phoenix, AZ, USA

## Abstract

**Background:**

Implementing multi‐site behavioral intervention trials in Alzheimer's disease (AD) has many unique challenges, leading to substantial variations in delivered intervention doses and cognitive findings. These issues can be addressed by the I**M**plementation Framework f**O**r **B**ehavioral and L**I**festy**L**e Interventions **I**n Al**Z**heimer's Diseas**E** (MOBILIZE) that was developed to guide the design and implementation of behavioral interventions in AD.

**Method:**

This study systematically evaluated the implementation outcomes of the 3‐site aerobic exercise and cognitive training (ACT) trial that employed MOBILIZE. Sites included University of Minnesota (UMN], University of Rochester (UR], and Arizona State University (ASU]. Building on the person‐centered care principle, MOBILIZE includes three implementation outcomes (screening, intervention adherence, and safety) with corresponding team processes to guide trial implementation. Screening was defined as time to complete the 4‐step screening phase and baseline testing. Intervention adherence included: attendance, session dose adherence, and days required to complete the prescribed 72 sessions. Exercise session dose was considered compliant if (1) the session's average heart rate or rating of perceived exertion was in prescribed zone of the session and (2) session length was met. Cognitive session dose was considered compliant if participant completed the prescribed length of cognitive training. Safety was operationalized as the number of study‐related adverse events (AEs).

**Result:**

The sample (*n* = 146) was 73.8±5.7 years in age and 23.4±2.1 on Montreal Cognitive Assessment score, with 48.0% female and 91.8% White. The median days of screening‐to‐enrollment averaged 98 days. For the entire sample, the mean average attendance was 76.7±28.6% (UMN: 79.7±32.3%; UR: 79.5±22.5%; ASU: 65.7±32.1%) (F=2.79, *p* = 0.07). Adherence to exercise session dose was 71.7±30.8% (UMN: 76.6±30.6%; UR: 70.5±30.5%; ASU: 60.1±30.1%) (F=1.98, *p* = 0.07). Adherence to cognitive session dose was 51.5±26.2% (UMN: 73.3±21.6%; UR: 39.6±32.8%; ASU: 35.7±20.3%) (F=21.24, *p* <0.01) respectively. The median length of the 6‐month intervention period was 188.0 (173.5‐197.3) days (UMN: 185.0 [170.0‐196.0] days; UR: 190.0 [182.0‐199.0] days; ASU: 184.0 [127.5‐207.5] days) (H(2)=4.50, *p* = 0.11). There were 10 study‐related adverse events and didn’t differ across sites (*p* = 0.76).

**Conclusion:**

MOBILIZE helped the ACT Trial achieve high intervention attendance and safety and may be particularly important for early‐stage and multi‐site trials in AD.

